# Revolutionizing Neurology: The Role of Artificial Intelligence in Advancing Diagnosis and Treatment

**DOI:** 10.7759/cureus.61706

**Published:** 2024-06-05

**Authors:** Meetali Kalani, Ashish Anjankar

**Affiliations:** 1 Biochemistry, Jawaharlal Nehru Medical College, Datta Meghe Institute of Higher Education and Research, Wardha, IND

**Keywords:** ai algorithms, brain signals, neuroimaging, brain-computer interfaces, precision medicine, neurological disorders, artificial intelligence

## Abstract

Artificial intelligence (AI) has emerged as a powerful tool in the field of neurology, significantly impacting the diagnosis and treatment of neurological disorders. Recent technological breakthroughs have given us access to a plethora of information relevant to many aspects of neurology. Neuroscience and AI share a long history of collaboration. Along with great potential, we encounter obstacles relating to data quality, ethics, and inherent difficulty in applying data science in healthcare. Neurological disorders pose intricate challenges due to their complex manifestations and variability. Automating image interpretation tasks, AI algorithms accurately identify brain structures and detect abnormalities. This accelerates diagnosis and reduces the workload on medical professionals. Treatment optimization benefits from AI simulations that model different scenarios and predict outcomes. These AI systems can currently perform many of the sophisticated perceptual and cognitive capacities of biological systems, such as object identification and decision making. Furthermore, AI is rapidly being used as a tool in neuroscience research, altering our understanding of brain functioning. It has the ability to revolutionize healthcare as we know it into a system in which humans and robots collaborate to deliver better care for our patients. Image analysis activities such as recognizing particular brain regions, calculating changes in brain volume over time, and detecting abnormalities in brain scans can be automated by AI systems. This lessens the strain on radiologists and neurologists while improving diagnostic accuracy and efficiency. It is now obvious that cutting-edge artificial intelligence models combined with high-quality clinical data will lead to enhanced prognostic and diagnostic models in neurological illness, permitting expert-level clinical decision aids across healthcare settings. In conclusion, AI's integration into neurology has revolutionized diagnosis, treatment, and research. As AI technologies advance, they promise to unravel the complexities of neurological disorders further, leading to improved patient care and quality of life. The symbiosis of AI and neurology offers a glimpse into a future where innovation and compassion converge to reshape neurological healthcare. This abstract provides a concise overview of the role of AI in neurology and its transformative potential.

## Introduction and background

In recent years, the field of neurology has witnessed a remarkable transformation thanks to the integration of artificial intelligence (AI) technologies. These advancements have revolutionized understanding, diagnosing, and treating neurological disorders. AI with its ability to analyze vast amounts of data and identify intricate patterns is making substantial contributions to neurology, ranging from early disease detection to personalized treatment strategies [1]. AI is concerned with creating computer algorithms that mimic human cognitive skills such as learning, reasoning, and self-correction. The growth of machine-based analysis of complex data for everyday clinical applications has shown tremendous progress [2]. This article explores AI's significant impact on neurology, highlighting key areas where AI is making a difference. AI has transcended its role as a technological advancement and has become a cornerstone of innovation across various industries [3]. In the realm of neurology, the integration of AI has ushered in a new era of possibilities, bringing about transformative changes in how we approach diagnosis, treatment, and research of neurological disorders [4]. AI techniques, particularly machine learning and deep learning algorithms have demonstrated remarkable capabilities in processing vast datasets, including medical images, genetic information, and patient records. These algorithms excel in detecting subtle patterns, aiding in the early diagnosis of conditions such as brain tumors, multiple sclerosis, and stroke. Precision medicine is a cornerstone of modern healthcare, and AI plays a pivotal role in tailoring treatments to individual patients [2]. AI algorithms identify genetic predispositions and predict treatment responses by analyzing genetic information. This personalized approach optimizes therapeutic interventions, enhancing patient outcomes. Brain-computer interfaces (BCIs) powered by AI provide novel communication and interaction for individuals with severe neurological disabilities. These interfaces interpret brain signals to control external devices, enabling paralyzed patients to regain some autonomy. AI's impact on neuroimaging analysis must be considered [1]. However, AI's ability to process vast amounts of data, recognize patterns and make data-driven predictions is redefining the landscape of neurology and yielding a plethora of benefits that were once thought to be unattainable [5]. Neurological illnesses ranging from Alzheimer's to epilepsy are so complicated that fresh techniques for understanding and controlling them are required. AI has proven to be a game changer, with its ability to extract insights from different datasets such as medical scans, genetic profiles, and patient histories [6]. The benefits of AI in neurology are far-reaching and multifaceted, extending across early diagnosis, personalized treatment, BCIs, neuroimaging analysis, treatment optimization, and even drug development [7]. As AI evolves and matures, its potential to revolutionize neurological healthcare becomes increasingly evident [8]. The benefits of AI in neurology are not just technological; they promise to enhance the lives of countless individuals affected by neurological disorders, ushering in an era of precision, efficiency, and improved quality of life [9].

Neurological disorders, characterized by their diverse manifestations and often enigmatic origins, have presented formidable challenges [10]. Integrating AI technologies into neurology has yielded many benefits, including early diagnosis, personalized treatment plans, neuroimaging analysis, treatment optimization, and groundbreaking research endeavors [1]. AI's prowess in sifting through medical records, deciphering complex images, and even predicting disease trajectories has become a cornerstone of modern medical practice. This article delves into the spectrum of AI technologies woven into the intricate tapestry of neurology [11]. AI's influence is undeniably pervasive, from the precision of diagnosing neurological conditions at their earliest stages to the tailoring of treatment strategies that resonate with individual patients. Beyond diagnosis and treatment, AI-driven BCIs empower individuals with severe neurological impairments, fostering a level of independence previously deemed unattainable [12]. As AI technologies continue to evolve, they hold the potential to illuminate the mysteries of the brain, unravel the complexities of neurological disorders, and expedite the discovery of novel therapies [4]. As we go into the future of precision medicine, we examine current deep-learning methodologies and ethical concepts in AI. 

## Review

Methodology

Search Strategy

A detailed search was done on PubMed and advanced Medical Subject Headings (MeSH) terms, such as “Artificial intelligence,” “neurology,” “advancement in neurology,” "diffusion tensor imaging," “new technologies,” "neurosciences," "neurosurgery," "ChatGPT," and "robotics," were used interchangeably and in combination.

Eligibility Criteria

The inclusion criteria consisted of all articles published between 2020 and 2022 discussing AI and neurology, were in English, and for which PubMed or the publisher provided open access. The excluded articles were in languages other than English, were not retrievable (i.e., not having open access), and discussed either AI or neurology but not both. A total of 70 articles were found, but only 42 of them were chosen to be included because it was determined that they were pertinent.

Inclusion and Exclusion Criteria

**Figure 1:**
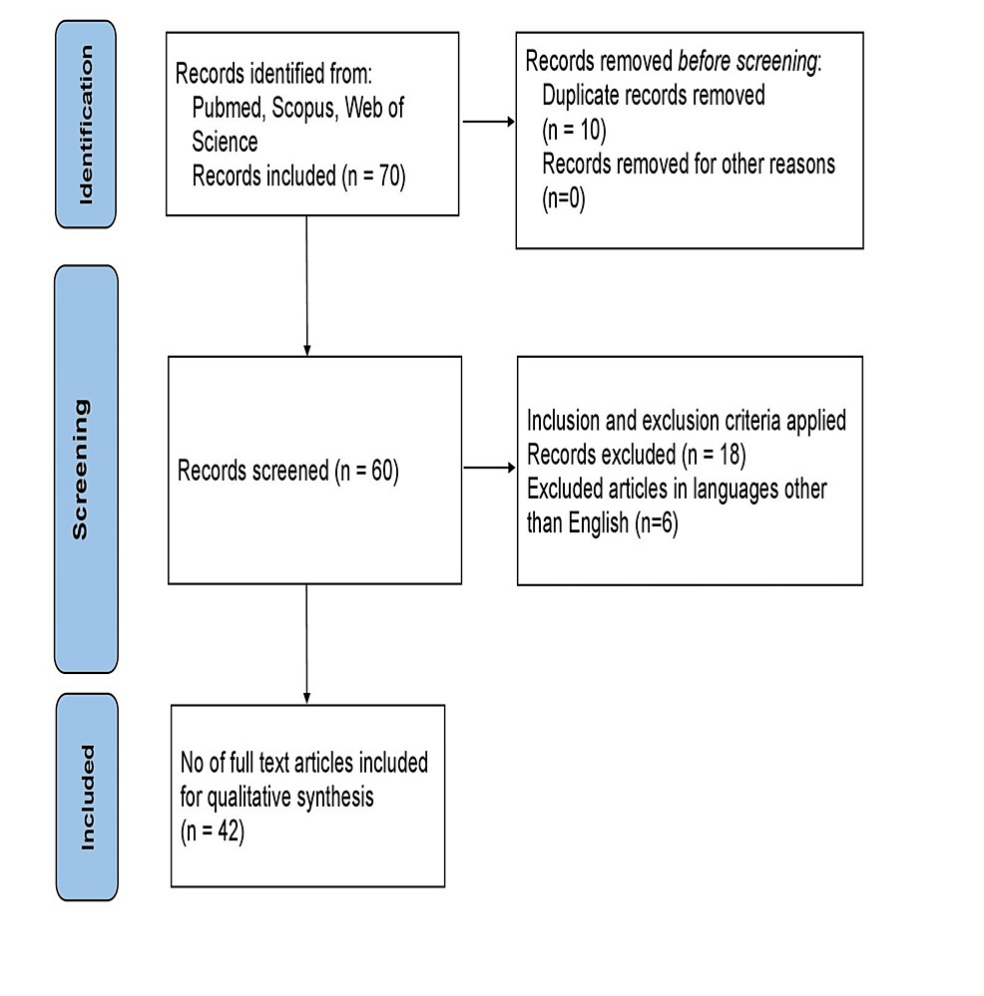
PRISMA (Preferred Reporting Items for Systematic Reviews and Meta-Analyses) flow diagram illustrates the process of study selection in the systemic review.

Records removed before screening were either due to duplication or for other reasons. During screening, inclusion, and exclusion criteria were also applied, from which 18 records were excluded and 6 records were in other languages except English. These were selected following the Preferred Reporting Items for Systematic Reviews and Meta-Analyses (PRISMA) guidelines [13]. A comprehensive outline of selection methods is given in Figure 1. 

Results

Neurological diseases are a diverse range of entities characterized by abnormal central and peripheral nervous system functions. Depending on the components engaged in the pathologic processes, they may exhibit various symptoms. Infections, traumas, lifestyle, and environmental factors are all potential causes of neurological disorders [14]. The acquisition and processing of greater and more sophisticated data has increased neuroscience's grasp of the nervous system's complexity. Today, numerous Neuroscience subfields, such as Computational Neuroscience, Neuroelectrophysiology, and Connectomics, use Big Data techniques to understand the structure and function of the brain. Neuroscience technology permits the collection of large, diverse data sets, the interpretation of which needs the creation of a new set of computational tools and resources for dealing with computationally challenging tasks [15]. Worldwide individuals need access to adequate healthcare due to infrastructural development. Intelligent technology in healthcare, known as digital healthcare, has resulted in significant advances in medical technology, such as clinical disease-supporting systems (CDSSs). Using CDSSs can give suitable diagnosis and treatment solutions to assist medical practitioners even when the problems are outside their expertise. The introduction of ChatGPT, among other advancements, is causing further changes in the healthcare environment, such as using AI in diagnosis and treatment [16].

Early diagnosis and prediction

One of the most critical aspects of treating neurological disorders is early diagnosis, which often remains challenging due to neurological symptoms' complex and subtle nature. AI algorithms can process extensive patient data, including medical histories, imaging results, and genetic information, to identify fine markers that human experts might overlook [7]. Transcranial Doppler (TCD) ultrasonography is a non-invasive approach for diagnosing cerebrovascular illness that employs the Doppler effect to identify the hemodynamic and physiological characteristics of the primary intracranial basilar arteries. It can give vital hemodynamic information that other diagnostic imaging modalities for cerebrovascular illness cannot assess [17]. Neurology's intricate web of disorders affecting the nervous system is undergoing a profound transformation through the integration of AI [18]. Early diagnosis is pivotal in neurology, as many conditions are progressive and irreversible. AI-powered algorithms analyze diverse patient data - medical history, imaging results, genetic profiles - to identify subtle markers of neurological disorders that might evade human observation [19]. In conditions like Alzheimer's or Parkinson's, AI can spot deviations long before symptoms manifest, enabling interventions at a more manageable stage. Neuroimaging is central to diagnosis [20]. AI excels at interpreting MRI, CT, and PET scans, identifying anomalies, quantifying brain structures, and tracking changes over time. These analyses expedite diagnosis and provide comprehensive insights, enhancing treatment planning [21].

Neuroimaging modalities

Neuroimaging encompasses various techniques, such as MRI, computed tomography (CT), positron emission tomography (PET), and single-photon emission computed tomography (SPECT). These modalities capture detailed structural, functional, and molecular information about the brain, enabling the visualization of abnormalities, connectivity patterns, and metabolic activity [22]. Interpreting complex neuroimaging data requires a high level of expertise and time-consuming analysis. This reduces the burden on radiologists and neurologists and enhances the accuracy and efficiency of diagnoses [23]. Neuroimaging, a cornerstone of modern neuroscience, has revolutionized our understanding of neurological disorders. Integrating AI technologies into neuroimaging practices has further elevated these tools' precision, efficiency, and diagnostic capabilities [24]. For instance, in stroke diagnosis, AI algorithms can swiftly analyze brain images to identify ischemic regions, guiding timely interventions [21].

Benefits of AI in neurology

AI offers numerous benefits to Neurology, revolutionizing how neurological disorders are diagnosed, treated, and understood. Some of the key benefits are mentioned in Table 1. 

**Table 1:** Benefits of artificial intelligence (AI) in neurology. MRI, magnetic resonance imaging; CT, computerized tomography Reference: open source.

Benefits	Description
Improved diagnosis	AI can analyze medical images (MRI and CT scans) and detect subtle abnormalities or early signs of neurological diseases with high accuracy, aiding in early diagnosis and intervention [21].
Enhanced disease prediction	Machine learning models can predict the risk of developing neurological disorders based on genetic, clinical, and lifestyle data, allowing for proactive preventive measures.
Personalized treatment plans	AI can analyze patient data to tailor treatment plans, considering individual factors like genetics and therapy response, optimizing treatment effectiveness.
Clinical decision support	Neurologists can benefit from AI-based decision support systems that provide evidence-based recommendations for diagnosis and treatment, reducing errors and improving patient outcomes.
Efficient data management	AI systems can manage and process vast amounts of patient data, making it easier for healthcare providers to access and organize information for informed decision-making.
Drug discovery	AI accelerates drug discovery by analyzing genetic data, simulating molecular interactions, and identifying potential drug candidates for neurological conditions, speeding up the research process.
Early intervention in neurodegenerative diseases	AI algorithms can track subtle changes in patient data over time, allowing for early intervention in conditions like Alzheimer's and Parkinson's, potentially slowing disease progression.
Telemedicine and Remote monitoring	AI-enabled telemedicine platforms and wearable devices facilitate remote monitoring of neurological patients, improving access to care and reducing the burden on healthcare facilities.

Evolution of BCIs 

BCIs bridge the gap between the human brain and external devices, enabling direct communication, control, and interaction. In a world where human-computer interaction is becoming increasingly integral to daily life, BCIs hold immense promise not only for those with physical disabilities but for society as a whole. BCIs have come a long way since their inception [25]. Early BCIs were primarily designed to assist individuals with severe motor impairments. These systems translated brain signals into commands for assistive devices such as computer cursors or robotic arms [26]. However, BCIs have evolved beyond mere assistive tools. They now encompass various categories.

Medical BCIs

These are used in the medical field for neurorehabilitation, allowing patients to regain lost motor functions and improve their quality of life [25].

Communication BCIs

BCIs have empowered individuals with locked-in syndrome or severe communication impairments to express their thoughts, facilitating social integration [27].

Entertainment and Gaming

BCIs have found applications in the gaming industry where players can control characters or devices using their thoughts [28].

Enhanced Cognitive Abilities

BCIs can augment human cognitive abilities, potentially enhancing memory, concentration, or problem-solving skills [29]. BCIs are already making a significant impact across multiple domains:

Assistive devices: BCIs enable paralyzed individuals to control wheelchairs, robotic limbs, or even type on computers, enhancing their autonomy [30].

Neurorehabilitation: In the medical field, BCIs aid stroke survivors and patients with spinal cord injuries in regaining lost functions through neurofeedback training [31].

Communication: BCIs offer a voice to individuals who were previously unable to communicate verbally, improving their quality of life [32].

Research: BCIs are indispensable tools in neuroscience, allowing researchers to understand brain function, study neurological disorders, and develop therapies [33].

Education: BCIs have educational potential, assisting in personalized learning and improving accessibility for students with disabilities [34]. BCIs transform how humans interact with technology, offering newfound abilities to those with disabilities and raising possibilities for enhancing human cognition. The ongoing development of BCIs will undoubtedly shape the future of medicine, communication, and human-computer interaction [25]. However, as these technologies advance, it is crucial to address the ethical, privacy, and accessibility concerns to ensure that the benefits of BCIs are accessible and safe for all, ultimately allowing society to harness their boundless potential for the betterment of humanity [35]. 

Advancing treatment strategies in neurology

Neurological disorders present complex challenges in diagnosis and treatment due to the intricate nature of the human brain. AI integration is revolutionizing treatment strategies in neurology by providing data-driven insights, personalized interventions, and improved clinical decision-making [36]. AI plays a pivotal role in the era of precision medicine. AI can identify individualized treatment options by analyzing extensive patient data, including genetic profiles, medical history, and treatment responses. This allows neurologists to tailor interventions based on a patient's unique genetic makeup and the specific characteristics of their condition. For example, AI can predict how a patient will respond to a particular medication or therapy, minimizing trial and error in treatment. AI-driven simulations and modelling enable the optimization of treatment plans [37]. These simulations consider multiple variables and potential outcomes to predict a given patient's most effective treatment strategies [38].

Based on previous patient data, AI can anticipate illness development. This allows neurologists to predict the progression of a neurological condition, change treatment strategies appropriately, and deliver more accurate prognoses to patients and their families. Predictive analytics can also help with long-term care planning [37]. AI can analyze patient data to assess the risk of disease progression or complications, considering individual factors and historical trends. Develop personalized treatment recommendations based on the patient's specific diagnosis, genetics, medical history, and other relevant factors. AI can help tailor treatment plans to individual needs [39].

AI can analyze drug interactions and predict potential adverse events, assisting clinicians to make informed decisions about medication choices. Implement AI-powered monitoring systems that track the patient's response to treatment, including the effectiveness of medications and any side effects [40]. Develop mobile apps or wearable devices enabling patients to track their symptoms in real time, with AI analyzing the data to provide insights to patients and healthcare providers [41].

AI-powered chatbots or apps can educate patients about their condition, treatment options, and lifestyle modifications, enhancing patient engagement and adherence to treatment plans. Prioritize patient preferences and quality of life when designing treatment plans, using AI to balance clinical efficacy and patient well-being [42]. AI can significantly enhance the quality and effectiveness of treatment plans for neurological disorders by leveraging data-driven insights and personalizing care for individual patients. However, the integration of AI should be done in collaboration with medical professionals to ensure that it aligns with the best practices and standards of neurology [37].

Challenges and considerations

Ensuring that AI models are robust, generalize well to diverse populations, and provide interpretable results is paramount [43]. Integrating AI into the field of neurology presents exciting opportunities, but it also comes with several challenges [1]. Obtaining high-quality and sufficiently large datasets for AI training and validation can be challenging, especially for rare neurological disorders. Integrating AI systems into healthcare IT infrastructure and ensuring compatibility with various data sources and formats can be complex [44]. Addressing ethical dilemmas related to patient consent, transparency, bias in AI algorithms, and responsible AI use is crucial. Navigating the evolving regulatory landscape for AI in healthcare, including obtaining approvals and ensuring compliance with changing regulations, poses challenges. Identifying and mitigating biases in AI algorithms to ensure fair and equitable diagnosis and treatment recommendations for diverse patient populations [45] and conducting rigorous clinical validation studies to demonstrate the real-world effectiveness and safety of AI-driven diagnostic and treatment tools. Encouraging patient engagement with AI-driven tools and, ensuring patients understand and trust AI recommendations, evaluating the cost-effectiveness of AI solutions in neurology and determining the return on investment for healthcare organizations [44]. Ensuring that AI-driven solutions prioritize patient outcomes, quality of life, and individual preferences [46]. Despite these challenges, the integration of AI in neurology holds immense promise for improving diagnosis, treatment, and patient care. Addressing these challenges will require ongoing collaboration, research, and a commitment to ethical and responsible AI use in healthcare [47].

Discussion

Neuroimaging has emerged as a critical tool in neurology, revolutionizing our understanding of the brain and its various disorders. The rapid development of neuroimaging techniques, along with AI and machine learning advancements, has led to remarkable progress in diagnosing, treating, and researching neurological conditions [48]. The history of neuroimaging can be traced back to the early 20th century when the discovery of X-rays paved the way for radiological Imaging of the brain. Over the decades, neuroimaging has evolved from simple X-rays to more sophisticated techniques, including CT, magnetic resonance imaging (MRI), PET, SPECT, and functional MRI (fMRI) [49]. For Stroke Diagnosis,* *CT and MRI are essential in identifying the type and location of a stroke, enabling rapid treatment decisions such as thrombolysis or clot retrieval [50]. Neuroimaging plays a crucial role in detecting brain tumors, allowing neurosurgeons to plan precise surgeries while minimizing damage to healthy tissue [51]. MRI is the primary tool for diagnosing and monitoring MS, visualizing lesions in the central nervous system that characterize the disease's progression [52]. Neuroimaging, including fMRI and PET scans, helps locate the epileptic focus, guiding surgical intervention when necessary [53]. PET and SPECT scans aid in diagnosing Alzheimer's, Parkinson's, and other neurodegenerative diseases by detecting specific biomarkers. Recent advancements in neuroimaging have expanded its capabilities and utility in neurology. Functional imaging, for example, enables the mapping of brain activity, aiding in the understanding of the neural basis of cognitive functions, emotions, and behaviors [54]. 

Diffusion tensor imaging (DTI) provides insights into white matter integrity and connectivity, aiding in studying conditions like traumatic brain injury and autism [55]. Magnetic resonance spectroscopy allows for the measurement of biochemical markers in the brain, aiding in diagnosing and monitoring conditions like brain tumors and metabolic disorders [56]. AI and machine learning algorithms can analyze large neuroimaging datasets to detect subtle abnormalities, predict disease progression, and assist in treatment planning with remarkable accuracy [21]. Neuroimaging has not only transformed diagnosis but also personalized treatment in neurology like high-resolution MRI and functional mapping help neurosurgeons plan minimally invasive surgeries, reducing patient recovery times and complications [57]. Targeted therapies aids in identifying specific brain regions affected by neurological conditions, allowing for targeted treatments like deep brain stimulation for Parkinson's disease [58]. By analyzing neuroimaging data alongside genetic and clinical information, neurologists can tailor treatment plans to each patient's unique profile [37]. While neuroimaging has brought significant advancements, challenges remain. These include ensuring access to advanced neuroimaging techniques, addressing ethical concerns related to data privacy, and developing more precise imaging modalities [59]. Looking ahead, neuroimaging in neurology is poised to continue its transformative journey, with emerging technologies like functional connectivity MRI, neuroimaging biomarkers, and real-time Imaging presenting exciting possibilities. AI's successful integration into neuroimaging also poses challenges. Data quality, privacy concerns, and ensuring AI transparency are crucial factors. As AI and machine learning evolve, integrating these technologies with neuroimaging promises more accurate diagnosis, better treatment outcomes, and a deeper understanding of the brain's intricacies [23]. AI holds tremendous potential in the field of neurology, from improving diagnostics to revolutionizing drug discovery and patient care. However, it is essential to address challenges related to data privacy, bias, clinical integration, ethics, and regulation to ensure that AI benefits patients while upholding high standards of healthcare. This data-driven approach guides physicians in making informed decisions and fine-tuning treatment strategies. Several AI-assisted brain computer/machine interface applications have been created to aid persons with neuromuscular illnesses such as cerebral palsy or spinal cord injuries. Furthermore, AI accelerates research and drug development in neurology. By swiftly analyzing molecular databases, AI identifies potential drug candidates and offers insights into disease mechanisms.

## Conclusions

AI is ushering in a new era of possibilities in neurology. From early diagnosis and personalized treatment to BCIs and drug discovery, AI drives transformative changes that enhance patient care and our understanding of neurological disorders. As AI technologies evolve, the synergy between technology and medical expertise promises to redefine the boundaries of neurological healthcare, offering improved patient outcomes and a brighter future for individuals grappling with neurological disorders. Through precise analysis of neuroimaging data, AI empowers clinicians and researchers to unlock deeper insights, enhance diagnostic accuracy, and pave the way for personalized treatment approaches that can positively impact the lives of individuals affected by neurological conditions. However, as these technologies advance, it is crucial to address the ethical, privacy, and accessibility concerns to ensure that the benefits of BCIs are accessible and safe for all, ultimately allowing society to harness their boundless potential for the betterment of humanity. The capabilities of AI extend to neuroimaging, where it automates complex image analysis tasks, enhances accuracy, and accelerates diagnoses. It optimizes treatment plans by simulating diverse scenarios, predicting outcomes, and reducing the margin for error in clinical decision-making. Furthermore, AI accelerates research and drug development, offering new hope for conditions that have long confounded medical science. In conclusion, AI has emerged as a vital partner in neurology, enhancing our ability to understand, diagnose, and treat neurological disorders with unparalleled precision and efficiency. As AI technologies continue to evolve, they promise to unravel the intricate mysteries of the human nervous system, leading to better care and improved quality of life for individuals affected by neurological conditions. The journey of AI in neurology is far from over; it is an evolving path toward a future where compassion and innovation converge to reshape the landscape of neurological healthcare.

## References

[1] Ahuja AS (2023). The impact of artificial intelligence in medicine on the future role of the physician. PeerJ.

[2] Li X, Zeng L, Lu X, Chen K, Yu M, Wang B, Zhao M (2023). A review of artificial intelligence in the rupture risk assessment of intracranial aneurysms: applications and challenges. Brain Sci.

[3] Basu K, Sinha R, Ong A, Basu T (2020). Artificial intelligence: how is it changing medical sciences and its future?. Indian J Dermatol.

[4] Patel UK, Anwar A, Saleem S (2021). Artificial intelligence as an emerging technology in the current care of neurological disorders. J Neurol.

[5] Bohr A, Memarzadeh K (2020). The rise of artificial intelligence in healthcare applications. AI Healthcare.

[6] Ahmed Z, Mohamed K, Zeeshan S, Dong X (2023). Artificial intelligence with multi-functional machine learning platform development for better healthcare and precision medicine. Database (Oxford).

[7] Singh KR, Dash S (2023). Early Detection of Neurological Diseases Using Machine Learning and Deep Learning Techniques: A Review. Artificial Intelligence for Neurological Disorders.

[8] Bhattacharya S (2022). Artificial intelligence, human intelligence, and the future of public health. AIMS Public Health.

[9] Harry A (2023). The future of medicine: harnessing the power of AI for revolutionizing healthcare. Int J Multidisip Res.

[10] Maldonado KA, Alsayouri K (2023). Physiology, Brain. StatPearls.

[11] Miller DD, Brown EW (2018). Artificial intelligence in medical practice: The question to the answer?. Am J Med.

[12] Yao Z, Wang H, Yan W, Wang Z, Zhang W, Wang Z, Zhang G (2023). Artificial intelligence-based diagnosis of Alzheimer's disease with brain MRI images. Eur J Radiol.

[13] Yao L, Zhang Y, Zhao C, Zhao F, Bai S (2022). The PRISMA 2020 statement: a system review of hospital preparedness for bioterrorism events. Int J Environ Res Public Health.

[14] Papadopoulou E, Pepe G, Konitsiotis S (2023). The evolution of comprehensive genetic analysis in neurology: implications for precision medicine. J Neurol Sci.

[15] Dipietro L, Gonzalez-Mego P, Ramos-Estebanez C, Zukowski LH, Mikkilineni R, Rushmore RJ, Wagner T (2023). The evolution of Big Data in neuroscience and neurology. J Big Data.

[16] Kim BJ (2023). The intersection of technology and humanity: exploring the ethics and potential of artificial intelligence in medicine. J Clin Neurol.

[17] Gan L, Yin X, Huang J, Jia B (2023). Transcranial Doppler analysis based on computer and artificial intelligence for acute cerebrovascular disease. Math Biosci Eng.

[18] Kumar Y, Koul A, Singla R, Ijaz MF (2023). Artificial intelligence in disease diagnosis: a systematic literature review, synthesizing framework and future research agenda. J Ambient Intell Humaniz Comput.

[19] Surianarayanan C, Lawrence JJ, Chelliah PR, Prakash E, Hewage C (2023). Convergence of artificial intelligence and neuroscience towards the diagnosis of neurological disorders—a scoping review. Sensors (Basel).

[20] Vrahatis AG, Skolariki K, Krokidis MG, Lazaros K, Exarchos TP, Vlamos P (2023). Revolutionising the early detection of Alzheimer's disease through non-invasive biomarkers: the role of artificial intelligence and deep learning. Sensors (Basel).

[21] Hosny A, Parmar C, Quackenbush J, Schwartz LH, Aerts HJ (2018). Artificial intelligence in radiology. Nat Rev Cancer.

[22] Hussain S, Mubeen I, Ullah N (2022). Modern diagnostic imaging technique applications and risk factors in the medical field: a review. Biomed Res Int.

[23] Monsour R, Dutta M, Mohamed AZ, Borkowski A, Viswanadhan NA (2022). Neuroimaging in the era of artificial intelligence: current applications. Fed Pract.

[24] Nielsen AN, Barch DM, Petersen SE, Schlaggar BL, Greene DJ (2020). Machine learning with neuroimaging: evaluating its applications in psychiatry. Biol Psychiatry Cogn Neurosci Neuroimaging.

[25] Shih JJ, Krusienski DJ, Wolpaw JR (2012). Brain-computer interfaces in medicine. Mayo Clin Proc.

[26] Lazarou I, Nikolopoulos S, Petrantonakis PC, Kompatsiaris I, Tsolaki M (2018). EEG-based brain-computer interfaces for communication and rehabilitation of people with motor impairment: a novel approach of the 21st century. Front Hum Neurosci.

[27] Rabbani Q, Milsap G, Crone NE (2023). The potential for a speech brain-computer interface using chronic electrocorticography. Neurotherapeutics.

[28] Frąckiewicz M (2023). The Advantages of Brain-Computer Interfaces for Gaming and Entertainment. https://ts2.space/en/the-advantages-of-brain-computer-interfaces-for-gaming-and-entertainment/?utm_medium=email&utm_source=transaction#.

[29] The benefits of brain-computer interfaces for cognitive enhancement (2023). The Benefits of Brain-Computer Interfaces for Cognitive Enhancement. September 11, 2023.

[30] Millán JD, Rupp R, Müller-Putz GR (2010). Combining brain-computer interfaces and assistive technologies: state-of-the-art and challenges. Front Neurosci.

[31] Bockbrader MA, Francisco G, Lee R, Olson J, Solinsky R, Boninger ML (2023). Brain computer interfaces in rehabilitation medicine. PM R.

[32] Young MJ, Lin DJ, Hochberg LR (2021). Brain-computer interfaces in neurorecovery and neurorehabilitation. Semin Neurol.

[33] Saha S, Mamun KA, Ahmed K (2023). Progress in brain computer interface: challenges and opportunities. Front Syst Neurosci.

[34] Frąckiewicz M (2023). The Benefits of Brain-Computer Interfaces for Education and Learning. https://ts2.space/en/the-benefits-of-brain-computer-interfaces-for-education-and-learning/?utm_medium=email&utm_source=transaction#.

[35] Burwell S, Sample M, Racine E (2023). Ethical aspects of brain computer interfaces: a scoping review. BMC Med Ethics.

[36] Segato A, Marzullo A, Calimeri F, De Momi E (2020). Artificial intelligence for brain diseases: a systematic review. APL Bioeng.

[37] Johnson KB, Wei WQ, Weeraratne D (2021). Precision medicine, AI, and the future of personalized health care. Clin Transl Sci.

[38] Chowdhury MZ, Turin TC (2020). Variable selection strategies and its importance in clinical prediction modelling. Fam Med Community Health.

[39] Vora LK, Gholap AD, Jetha K, Thakur RR, Solanki HK, Chavda VP (2023). Artificial intelligence in pharmaceutical technology and drug delivery design. Pharmaceutics.

[40] Shaik T, Tao X, Higgins N, Li L, Gururajan R, Zhou X, Acharya UR (2023). Remote patient monitoring using artificial intelligence: current state, applications, and challenges. Wiley Interdiscip Rev Data Min Knowl Discov.

[41] Jadczyk T, Wojakowski W, Tendera M, Henry TD, Egnaczyk G, Shreenivas S (2023). Artificial intelligence can improve patient management at the time of a pandemic: The role of voice technology. J Med Internet Res.

[42] Aldoseri A, Al-Khalifa KN, Hamouda AM (2023). Re-thinking data strategy and integration for artificial intelligence: concepts, opportunities, and challenge. Appl Sci.

[43] Bajwa J, Munir U, Nori A, Williams B (2023). Artificial intelligence in healthcare: transforming the practice of medicine. Future Healthc J.

[44] Gerke S, Minssen T, Cohen G (2020). Ethical and legal challenges of artificial intelligence-driven healthcare. Artif Intell Healthcare.

[45] Lee D, Yoon SN (2021). Application of artificial intelligence-based technologies in the healthcare industry: opportunities and challenges. Int J Environ Res Public Health.

[46] Davenport T, Kalakota R (2019). The potential for artificial intelligence in healthcare. Future Healthc J.

[47] Ranson JM, Bucholc M, Lyall D (2023). Harnessing the potential of machine learning and artificial intelligence for dementia research. Brain Inform.

[48] Mishra SK, Singh P (2010). History of neuroimaging: the legacy of William Oldendorf. J Child Neurol.

[49] Birenbaum D, Bancroft LW, Felsberg GJ (2011). Imaging in acute stroke. West J Emerg Med.

[50] Cole KL, Findlay MC, Kundu M, Johansen C, Rawanduzy C, Lucke-Wold B (2023). The role of advanced imaging in neurosurgical diagnosis. J Mod Med Imag.

[51] Kaunzner UW, Gauthier SA (2017). MRI in the assessment and monitoring of multiple sclerosis: an update on best practice. Ther Adv Neurol Disord.

[52] Duncan JS, Winston GP, Koepp MJ, Ourselin S (2016). Brain imaging in the assessment for epilepsy surgery. Lancet Neurol.

[53] Zhu L, Ploessl K, Kung HF (2014). PET/SPECT imaging agents for neurodegenerative diseases. Chem Soc Rev.

[54] Glover GH (2011). Overview of functional magnetic resonance imaging. Neurosurg Clin N Am.

[55] Hamsini BC, Reddy BN, Kumaran SN and SP, Hamsini BC, Reddy BN, Kumaran SN and SP (2018). Clinical application of MR spectroscopy in identifying biochemical composition of the intracranial pathologies. IntechOpen.

[56] Jolesz FA (2009). MRI-guided focused ultrasound surgery. Annu Rev Med.

[57] Dormont D, Seidenwurm D, Galanaud D, Cornu P, Yelnik J, Bardinet E (2010). Neuroimaging and deep brain stimulation. AJNR Am J Neuroradiol.

[58] White T, Blok E, Calhoun VD (2023). Data sharing and privacy issues in neuroimaging research: opportunities, obstacles, challenges, and monsters under the bed. Hum Brain Mapp.

[59] Pope WB, Djoukhadar I, Jackson A (2016). Neuroimaging. Handb Clin Neurol.

